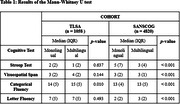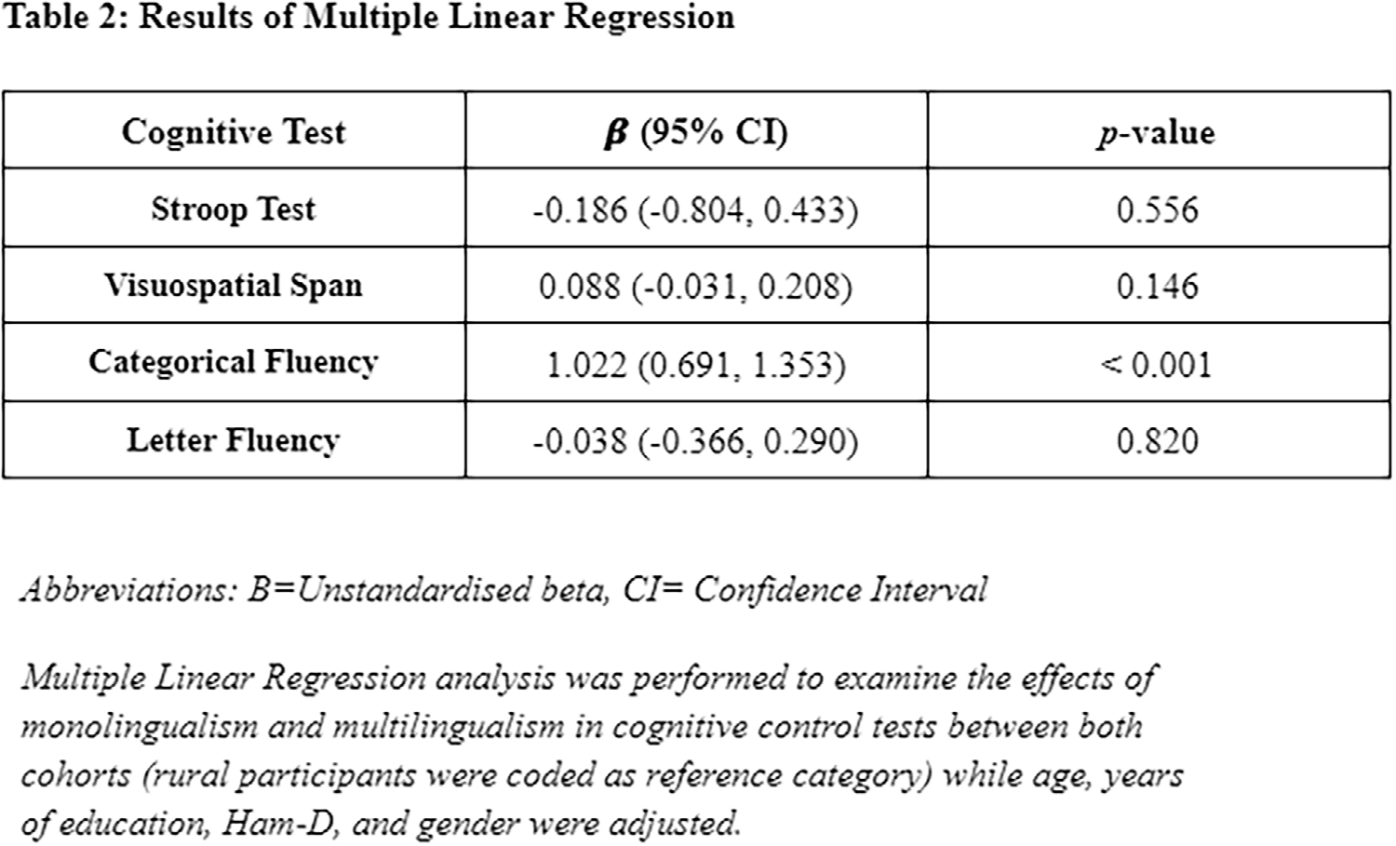# Language and Cognitive Control in Aging Urban and Rural Cohorts in India

**DOI:** 10.1002/alz.091241

**Published:** 2025-01-03

**Authors:** Meenakshi Menon, Palash K Malo, Abhishek Mensegere Lingegodwa, Albert Stezin, Shafeeq K Shahul Hameed, Rajitha Narayanasamy, Vindhya Vishwanath, Meghana R, Divya N M, Ajith Partha, Amitha C M, Dev Kumar HS, Prathima Arvind, Deepashri Agrawal, Sunitha HS, Goutham Velavarajan, Banashree Mondal, Jonas S. Sundarakumar, Thomas Gregor Issac

**Affiliations:** ^1^ Centre for Brain Research, Indian Institute of Science, Bangalore, Karnataka India

## Abstract

**Background:**

Cognitive control deficits can be early indicators of cognitive decline in individuals. Studies have found a bilingual advantage in cognitive control, however, there is little research on the Indian population, particularly those residing in rural areas. We aimed to investigate how cognitive control is influenced by the number of languages known to individuals among the aging population in both urban and rural settings in South India.

**Method:**

The study comprises cross‐sectional data from healthy individuals aged 45 years and above, with 1058 participants from the urban Tata Longitudinal Study on Ageing (CBR‐TLSA) cohort and 4820 participants from the rural Srinivaspura Ageing, Neuro Senescence, and Cognition (CBR‐SANSCOG) study cohort. Both study protocols are harmonized. Cognitive control was assessed using the Stroop, visuospatial span, categorical fluency, and letter fluency tests. Mann‐Whitney U test was used to compare monolingual and multilingual participants’ performance in cognitive control tasks separately. The differences in monolingualism and multilingualism on cognitive control were adjusted with age, gender, years of education, cohort, and Hamilton Depression Rating Scale (Ham‐D) scores using the General Linear Model.

**Result:**

Urban participants performed better than their rural counterparts in all four cognitive control tasks (Table 1). Within the urban cohort, multilingual participants’ performed better than the monolingual participants in the categorical fluency task, whereas, in the rural cohort, multilingual participants performed better in all four tasks. Monolingual and multilingual participants in the urban cohort performed better than their respective counterparts in the rural cohort in the cognitive control tasks. After adjusting for the covariates, we found a significant difference between monolingual and multilingual participants on the categorical fluency task, with multilingual participants performing better than monolinguals (Table 2).

**Conclusion:**

Our results indicated a multilingual advantage in cognitive control in aging Indians.